# Health Risk Assessment of Heavy Metals in Traditional Cosmetics Sold in Tunisian Local Markets

**DOI:** 10.1155/2016/6296458

**Published:** 2016-02-22

**Authors:** Mohamed Anouar Nouioui, Salah Mahjoubi, Asma Ghorbel, Marouen Ben Haj Yahia, Dorra Amira, Hayet Ghorbel, Abderrazek Hedhili

**Affiliations:** ^1^Centre d'Assistance Médicale et Urgente CAMU, 1008 Tunis Cedex, Tunisia; ^2^Laboratoire de Toxicologie et Environnement LR12SP07, 10 rue Abou Kacem Chabbi, Montfleury, 1008 Tunis Cedex, Tunisia; ^3^Laboratoires de Pharmaceutiques, Cosmétiques et Détergents, Centre Technique de la Chimie, 12 rue de l'Usine, Charguia II, Carthage, 2035 Tunis, Tunisia

## Abstract

This study was undertaken in order to determine heavy metal contents in twelve (*n* = 12) henna brands and eleven (*n* = 11) kohl products. An analytical test was performed for Pb, Cd, Cu, and Zn in henna and kohl products using atomic absorption spectrophotometery. The overall mean concentrations of heavy metals in henna varied between 1.2 and 8.9 *μ*g g^−1^ for Pb; 0.8 and 18.6 *μ*g g^−1^ for Cd; 0.5 *μ*g g^−1^ and 3.3 *μ*g g^−1^ for Cu; and 3.7 *μ*g g^−1^ and 90.0 *μ*g g^−1^ for Zn. As for kohl products, Pb concentrations ranged between 51.1 *μ*g g^−1^ and 4839.5 *μ*g g^−1^, Cd concentrations ranged between 1.0 *μ*g g^−1^ and 158.6 *μ*g g^−1^, Cu concentrations ranged between 2.5 *μ*g g^−1^ and 162.5 *μ*g g^−1^, and Zn concentrations ranged between 0.7 *μ*g g^−1^ and 185.0 *μ*g g^−1^. The results of our study revealed that Pb, Cd, Cu, and Zn contents in investigated samples were high, making from the prolonged use of such products a potential threat to human health. Therefore, major quality controls are recommended in order to enforce acceptable limits of potential contaminants in cosmetics and good manufacturing practice.

## 1. Introduction

Personal care products and facial cosmetics are commonly used by millions of consumers on a daily basis. Direct application of cosmetics on human skin makes it vulnerable to a wide variety of ingredients. Despite the protecting role of skin against exogenous contaminants, some of the ingredients in cosmetic products are able to penetrate the skin and to produce systemic exposure [1–4].

Natural and synthetic substances may produce local effects on human skin, such as irritation, sensitization, allergy, or photoreactions [3, 5]. Among the hazardous substances contained in cosmetics, heavy metals are widely diffused in colored make-up products.

Henna and kohl are typical examples of traditional cosmetics widely used in Middle East and North Africa. Henna “*Lawsonia inermis*” is a flowering plant and the sole species of the* Lawsonia* genus. Its powder has been used since antiquity to dye skin, hair, and fingernails, as well as fabrics including silk, wool, and leather. Kohl is a powder traditionally made by grinding galena and other ingredients to darken the eyelids or as a mascara for the eyelashes [6].

These two products may contain relatively large amounts of heavy metals present in ingredients or accidently introduced during the preparation steps.

In fact, kohl or surma as eye cosmetic has been identified as a suspected source of Pb exposure to the ocular system in a number of adults and children [7–9]. It is composed mainly of galena (PbS), amorphous carbon, zincite (ZnO), sassolite (H_3_BO_3_), minium (Pb_3_O_4_), magnetite (Fe_3_O_4_), goethite (FeO(OH)), cuprite (Cu_2_O), and talc (Mg_3_Si_4_O_10_(OH)_2_) [10–14]. Because of its composition, kohl is considered by the US Food and Drug Administration (FDA) as unsafe for use and as an illegal substance to be imported or sold in the United States [15, 16] while, in other countries such as Tunisia, it is still largely sold in markets without any legal control.

Henna cultivated in contaminated zones may also be considered as a potential source of poisoning by heavy metals and its daily use may increase the body intake on heavy metals through percutaneous absorption. Besides, the excipients used with henna to fix the color may contain p-phenylenediamine (PPD), a toxic organic substance derivative of aniline which is responsible for many serious health problems.

For an accurate determination of heavy metal contents in cosmetic products, a variety of analytical techniques have been used for the assessment of metals in cosmetics, such as Laser Induced Breakdown Spectroscopy (LIBS) [17], Inductively Coupled Plasma Mass Spectrometry (ICP-MS) [18], Inductively Coupled Plasma-Optical Emission Spectrometry (ICP-OES) [19], Flame Atomic Absorption Spectrometry (FAAS) [4, 20], and Graphite Furnace Atomic Absorption Spectrometry (GFAAS) [21].

The high risk of heavy metal contamination by traditional locally sourced cosmetics sold in Tunisian markets in the absence of any legal control such as henna and kohl requires a thorough examination of their contents of trace metals.

Therefore, the objective of the present study was to examine the content of henna and kohl samples commonly sold in Tunisian markets.

## 2. Materials and Methods

### 2.1. Study Design

The study was carried out by the laboratory of toxicology and environment. Samples were collected from a well-known local open market in Tunis, Tunisia, where it is possible to buy inexpensive cosmetic products. At these shops, personal care products imported from different countries as well as those locally manufactured are sold. The target samples were only kohl and henna products because they are widely used among the Tunisian population and did not include high quality brand cosmetic products that are generally sold in beauty stores or pharmacies. The sampling was designed to ensure a maximum number of different brands of henna and kohl products. Every kohl and henna brand sold in the market was analyzed at least in triplicate. Keeping in view their daily use and their possible health impacts, twelve henna brands and eleven kohl products were purchased to assess their contents of heavy metals. Among the selected henna brands, six brands were imported from Sudan, India, Lybia, and Pakistan and the rest were locally manufactured. Concerning kohl samples, nine brands were imported from India, Pakistan, China, Saudi Arabia, and France and five were local brands. Samples were stored at room temperature until analysis.

### 2.2. Reagents and Standards

Analytical grade nitric acid 65% (w/v), perchloric acid 70% (w/v), and sulfuric acid 96% (w/v) purchased from Merck (Darmstadt, Germany) were used for sample preparation. Aqueous calibration solutions of Pb, Cd, Zn, and Cu were prepared by appropriate dilutions of certified stock solutions (1000 mg L^−1^) purchased from Wako (Wako, Japan). Plastic bottles, polyethylene tubes, autosampler cups, and glassware were soaked in 10% (v/v) HNO_3_ for 24 h, rinsed with Milli-Q water, dried, and stored in a closed polypropylene container.

### 2.3. Sample Digestion

Henna samples were wetly digested following the recommendations of the International Atomic Energy Agency (IAEA) [22] and according to the procedure reported by Lekouch et al. [23] with slight modifications. Henna samples were oven-dried overnight at 70°C to constant weight and then stored in desiccators. 0.2 g of each of the dried samples was accurately weighed in Teflon tubes. 5 mL of nitric acid was added and the samples were kept for 1 h at room temperature and then placed in aluminium block on hot plate at 90°C and digested for 3 h. The digests were cooled and filtered with Whatman number 42 and were diluted up to the mark (50 mL) into a calibrated flask.

Kohl samples were wetly digested with 2 : 1 : 1 mixture of nitric acid (65%), sulfuric acid (96%), and perchloric acid (70%) [23] on a hot plate in fuming hood near to dryness [24] by increasing the temperature for 2-3 h. The solutions were left to be cooled and filtered into a calibrated flask (50 mL) by Whatman number 42 and were diluted up to the mark.

### 2.4. Sample Analysis

Precise determination of heavy metals content in cosmetic products is quite important because there is a narrow margin of safety between adequate amount and overconsumption. Among the various methods suggested for heavy metals analysis in cosmetic products, atomic absorption spectrometry was the most frequently used because of its relative simplicity, lower cost, and low sample volume requirements and its low detection limits.

Due to the unavailability of certified reference material for metal analysis in kohl and henna, the accuracy of the method was determined by measuring the recovery of analytes. Spiked henna and kohl samples were run with the test samples and blanks using the same procedure. The analytical recovery for Pb, Cd, Zn, and Cu at concentrations tested (0.02 and 0.1 *μ*g mL^−1^) ranged between 95 and 105%.

#### 2.4.1. Graphite Furnace Atomic Absorption Spectrophotometry GFAAS

Pb and Cd concentrations were detected by Shimadzu AA 6800 atomic absorption spectrophotometer (Shimadzu, Kyoto, Japan) equipped with GFA-EX7 graphite furnace atomizer and ASC 6100 Autosampler (Shimadzu, Kyoto, Japan). Atomization was made on pyrolytic coated graphite tubes from Shimadzu. Nonspecific absorption of radiation was corrected using deuterium background correction. Integrated absorbance (peak area) was used for signal evaluation.

Pb and Cd hollow cathode lamps from Hamamatsu Photonics (Kyoto, Japan), operating at 10 and 8 mA, respectively, were used as radiation sources. The absorbance was read at 283.3 and 228.8 nm, respectively. Argon (99.999%) (Air Liquid, Tunis, Tunisia) was used as the purge gas. The furnace heating program is shown in Table 1. The absorbance was measured three times. Calibration standards were prepared using standard addition method. Each digested samples were divided into six equal portions to whom known amount of aqueous Pb and Cd concentrations were added to give final concentrations which covered the range 0 to 0.1 *μ*g mL^−1^ and 0 to 0.008 *μ*g mL^−1^ for Cd. Dilution correction was applied for samples diluted or concentrated during analysis.

#### 2.4.2. Flame Atomic Absorption Spectrophotometry

Analyses of kohl sample were carried out on a flame spectrophotometer (Perkin Elmer 308 B) for Cu and Zn. The absorbance was read at 324.7 nm and 213.9 nm at 0.7 nm slit width. The calibration curves were linear up to 5 *μ*g mL^−1^ for Cu and 1 *μ*g mL^−1^ for Zn. The gas flow used for the flame analysis was as follows: air, 13.5 L min^−1^; and acetylene, 2 L min^−1^.

#### 2.4.3. Energy Dispersive X-Ray Fluorescence Spectrometry EDXRF

For EDXRF analysis, samples were necessarily ground to a powder and then mounted in sample cups. Diffraction data were collected using Bruker S2 Ranger spectrometer with XFlash® Silicon Drift Detector and 50 KV Pd X-Ray Tube. These data were used to determine the elemental composition present in samples. The detection was qualitative and the elemental range covered all elements from Na to U. A bright metallic kohl in rock form identified as galena or lead sulfide was also analyzed for comparison with local kohl product.

## 3. Results and Discussions

In this study, twelve henna brands and eleven kohl (surma) products purchased from local Tunisian markets were analyzed for assessment of Pb, Cd, Cu, and Zn contents. Results were summarized in Table 2.

### 3.1. Heavy Metal Contents in Kohl Samples

The overall (*n* = 11) mean concentrations of analyzed heavy metals were as follows: 1926.9 ± 70.4 *μ*g g^−1^ (range: 54.1–4839.5 *μ*g g^−1^) with the highest concentration in surma “S-10” and the lowest in surma “S-9” for Pb; 59.5 ± 1.9 *μ*g g^−1^ (range: 0.0–158.6 *μ*g g^−1^) with the highest concentration in surma “S-10” and minimum (not detectable) in surma “S-9” for Cd; 80.4 ± 0.5 *μ*g g^−1^ (range: 0.7–185 *μ*g g^−1^) with the highest concentration observed in surma “S-10” followed by the lowest concentration in surma “S-9” for Zn; and 43.9 ± 0.3 *μ*g g^−1^ (range: 2.5–162.5 *μ*g g^−1^) with the highest concentration in surma “S-3” and the lowest concentration in surma “S-4” for Cu.

In order to establish the predominant contaminant in the analyzed kohl samples, the mean heavy metal contents were arranged in decreasing order. The result was the following: Pb > Zn > Cd > Cu. Thus, in analyzed samples, Pb was the most predominant element. Its concentration was much higher than all the other investigated metals. This finding was also reported by Ullah et al. [25] for different brands of kohl purchased from a local market in Pakistan. All the analyzed kohl products contained more Cu than Cd while, in the present study, the content of Cu was slightly lower than Cd.

Purchased samples were also divided into two groups. The first group entailed all the products which were manufactured in Tunisia while the second group included all imported items. Many of the local marketed kohl products were “home-made” products. These products were prepared according to local traditions by herbalists or unauthorized companies and sold in markets unlabeled and without proper packing. The traditional recipes of kohl may include the incorporation of various herbs like pepper, musk, and ginger.

All foreign kohl brands were imported from eastern countries including India, Pakistan, China, and Saudi Arabia with the exception of kohl S-9 which was manufactured in France.

Both groups contained considerable amounts of heavy metals and showed a wide variation among the samples. The overall mean concentrations of Pb in local kohl samples were 2020 ± 90.9 *μ*g g^−1^ versus 1849.3 ± 53.2 *μ*g g^−1^ in imported products.

The overall mean concentrations of Cd in local kohl products were 71.1 ± 2.9 *μ*g g^−1^ versus 47.9 ± 2.4 *μ*g g^−1^ in foreign brands. For Zn and Cu contents in local brands, the overall mean concentrations were 76.6 ± 0.4 *μ*g g^−1^ and 87.0 ± 0.5 *μ*g g^−1^ versus 83.6 ± 0.6 *μ*g g^−1^ and 8.0 ± 0.2 *μ*g g^−1^, respectively, in imported brands. With the exception of Zn content, all the local brands revealed higher concentrations of Pb, Cd, and Cu.

These findings were predictable since as previously mentioned, many of the local products were home-made prepared without precautions against contamination by heavy metals. These “home-made” kohl samples are often prepared by grinding galena (lead sulfide) instead of amorphous carbon or organic charcoal. Plant oils and the soot from various nuts, seeds, and gum resins are often added. Unfortunately, the nonlead products are considered to be of inferior quality to the older traditional varieties and, therefore, there has been an increase in the use of handmade, lead-based kohl.

The lowest concentrations of Pb and Cd were in sample S-9 manufactured in France while the highest concentrations were in sample S-10 manufactured from India. Although the number of brands was small for any statistical comparison, the difference in terms of Pb, Cd, and Zn contents between the product imported from France and all other products including the locally manufactured ones was remarkable. This difference questions the way of preparing the kohl products and the nature of ingredients used. Unfortunately, due to the small number of samples in the present case, this matter could not be investigated. However, this problem has been raised by a Danish survey conducted on different brands of commercialized kohl in Denmark. In all the samples investigated, the highest concentration was measured in product from India [26]. According to the authors of the survey, the difference was attributed to the way of manufacturing kohl between “eastern” and “western” countries and the lack of good manufacturing practice [26]. The elemental composition of one lead-based local kohl sample (S-5) was investigated by energy dispersive X-ray fluorescence (EDXRF) and compared to that of unprocessed natural stone “hajar” kohl product sold in the market.

**Figure 1:**
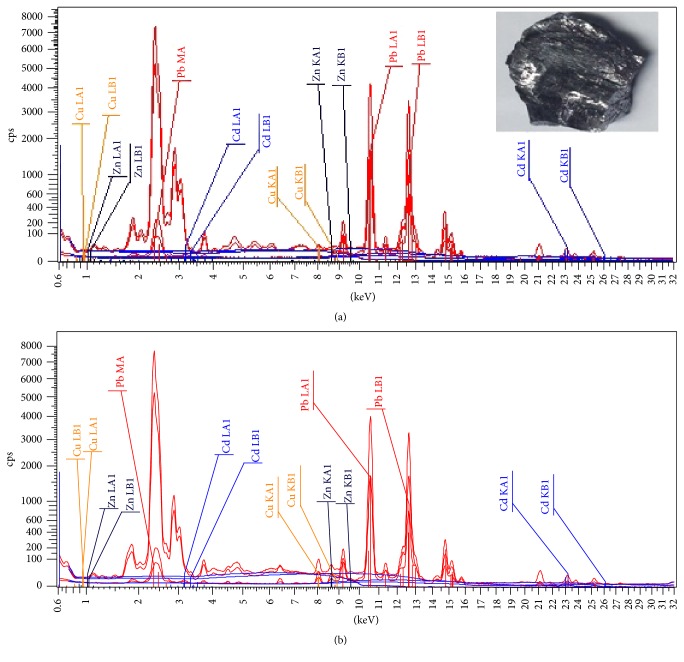
EDXRF spectra of both (a) the natural stone kohl sample and (b) the local kohl sample (S-5).

The EDXRF analysis of the natural stone kohl sample revealed Pb levels in excess of 96.59% of product weight (Table 3). The lustre and high Pb content as well as the presence of sulfur are consistent with its traditional identity as galena or Pb sulfide (Figure 1(a)). As for the local home-made kohl sample (S-5), the comparison to the spectra of the kohl stone revealed that this home-made kohl powder consisted predominantly of Pb (94.09%) explaining its high Pb concentration (2745 *μ*g g^−1^). Zinc and copper were also identified at levels of 0.23% and 0.38% of the product weight, respectively. Other elements including sulfur, calcium, chlorine, and silicon were also present at low concentrations (Table 3).

Comparison between literature and current study showed that the obtained Pb concentrations were close to those found in kohl samples from Middle East and Asia. Ullah et al. [25] conducted a study to determine heavy metal contents by FAAS in 15 cosmetic products both imported and locally manufactured by unauthorized company marketed at district Kohat, in Pakistan. The investigated products included three nonmedicated shampoos, three talc powders, three lipsticks, three kohl samples, and three cream samples. The highest contents for Pb were found in two kohl samples with overall mean concentrations of 1071 *μ*g g^−1^ and 1005 *μ*g g^−1^. Al-Ashban et al. [27] analyzed Pb along with Al and Sb in a total of 107 kohl samples (branded and unbranded) collected from different regions of Saudi Arabia by atomic absorption spectrometry. The analyzed kohl samples originated from commercial manufacturers from Saudi Arabia, Pakistan, India, and Iran. The authors stated that about 35% of kohl products sold were prepared by kohl sellers themselves and most of this kohl was sold without proper labels and packing while 65% of kohl sellers obtained properly labelled kohl from different sources. Pb levels up to 53% (w/w) of the product (530000 *μ*g g^−1^) were detected in some kohl preparations. Maximum Al and Sb concentrations were found to be 0.557 and 0.21%, respectively. Some samples were found to contain camphor and menthol. The blood of 20 regular kohl users was also analyzed and results showed high Pb concentration and relatively low haemoglobin levels. High Pb contents were also reported by Lekouch et al. [23] in 10 kohl samples obtained from local herbalists in the Marrakech markets. Four kohl samples were originated from Yemen, Algeria, Sudan, and Saudi Arabia and the rest were local products. Pb concentrations were extremely high in all types of analyzed kohl. Local kohl was particularly charged with Pb (89% w/w) especially the kohl composed of pure galena. Lower concentrations were found in some types of “spiced” kohl where kohl is mixed with various herbs and animal and mineral products. The authors explained this difference by the fact that the addition of many excipients dilutes the concentration of Pb in kohl. The elemental composition of 21 kohl specimens consisting of 6 home-made powders, 9 commercial preparations, and 6 natural stone kohl samples originating from various parts of Saudi Arabia, India, and the Middle East was investigated by Al-Hazzaa and Krahn [28] using energy dispersive X-ray analysis (EDXRF). The data revealed the presence of significant Pb levels in two-thirds of the kohl specimens analyzed at concentrations ranging from 2.9 to 100% w/w (mean 48.5%). Other elements were present in kohl preparations including Al, C, Fe, Ti, Ca, Mg, O, Ag, Si, S, and Sb. Only 7 kohl specimens were totally lead-free, 4 had Pb contents in the range of 2.9%–34.1% w/w, and 10 had lead levels in excess of 84%. Carbon levels in excess of 60% were detected in six kohl samples and Sb was present in only one kohl specimen at a concentration of 7.8%. Hardy et al. [10] reported that, among 18 kohl samples purchased from Cairo and analyzed using X-ray powder diffraction (XRPD) and scanning electron microscopy (SEM), the main component of six samples (4 originated from Egypt and 2 from India) was found to be galena (PbS). For a further ten samples the main component was found to be one of the following: amorphous carbon, calcite (CaCO_3_), cuprite (Cu_2_O), goethite (FeO(OH)), and elemental silicon or talc (Mg_3_Si_4_O_10_(OH)_2_). As For the last two samples, the main component of each was an unknown amorphous organic compound. Besides, in a previous work conducted on 23 kohl samples obtained in the United Arab Emirates (19 from Abu Dhabi and 4 from Dubai) using X-ray powder diffraction (XRPD) and scanning electron microscopy (SEM), Hardy et al. [12] reported that the main component found in 11 samples was galena (PbS) while, for the remaining 12 samples, the main component was found to be one of the following: amorphous carbon, zincite (ZnO), sassolite (H_3_BO_3_), or calcite/aragonite (CaCO_3_). Pb was also detected in 26 brands of lipsticks and 8 different brands of eye shadows marketed in Saudi Arabia. The mean lead concentrations in lipsticks samples were in the range of 0.27–3760 *μ*g g^−1^ wet weight. Four brands of lipsticks had Pb concentration above 20 *μ*g g^−1^ and three brands from China with mean concentrations of 2720.5 *μ*g g^−1^, 2522.5 *μ*g g^−1^ and 3760 *μ*g g^−1^ wet weight. As for Pb content in pressed powder eye shadow, the mean concentrations ranged from 0.42 to 58.7 *μ*g g^−1^ wet weight with only one brand with Pb content exceeding 20 *μ*g g^−1^ [21]. Lower Pb contents have also been reported in the literature. In A Danish survey conducted on 18 samples of kohl and 17 samples of henna purchased from retail shops and ethnic shops and analyzed by ICP-MS, Pb was found in 10 products at a mean concentration ranging from 0.30 to 1 *μ*g g^−1^, and in 4 products at a concentration ranging from 1 to 4 *μ*g g^−1^. Pb was only found at high concentration (280 *μ*g g^−1^) in one kohl product that was manufactured in India [26].

Volpe et al. [4] evaluated the content of Pb in 20 samples of eye shadows marketed in a local open market and in a franchise store in Benevento, Italy. The content of Pb was measured by FAAS while the quantification of Cd, Co, Cr, and Ni was performed by ICP-MS. Mean Pb concentrations ranged between 0.25 *μ*g g^−1^ and 81.50 *μ*g g^−1^. The highest values corresponded to eye shadows imported from China.

As for Cd content in kohl, the results of our study were higher than those reported by Volpe et al. [4] for Cd content in eye shadow where Cd mean levels ranged from 0.6 ng g^−1^ to 33.4 ng g^−1^. The mean Cd levels reported by Ullah et al. [25] in kohl samples varied from 0.095 to 0.942 *μ*g g^−1^ and only three products among the 18 kohl samples analyzed by the Danish survey contained Cd at concentrations of 0.06 *μ*g g^−1^, 0.08 *μ*g g^−1^, and 1.8 *μ*g g^−1^ [26]. For Cu content in kohl, the highest amount found was 162.5 *μ*g g^−1^ in a local brand (S-3) with an overall mean concentration of 43.9 *μ*g g^−1^ for all the analyzed 11 kohl samples. This value was approximately two times lower than the overall mean concentration reported by Ullah et al. [25] for the 3 tested kohl samples (104.57 *μ*g g^−1^) in which the highest Cu amount was found in kohl manufactured in Pakistan (302.2 *μ*g g^−1^). Copper was also found at a concentration of 950 *μ*g g^−1^ in a kohl sample manufactured from India [26].

The highest Zn content in the 11 kohl samples analyzed was 185 *μ*g g^−1^ in kohl from India with an overall mean content of 80.4 *μ*g g^−1^. This result was two times lower than the overall mean Zn content found (254.55 *μ*g g^−1^) by Ullah et al. [25] for the three kohl products from Pakistan. The reported values were 1.362 *μ*g g^−1^, 253.5 *μ*g g^−1^, and 508.8 *μ*g g^−1^. Higher amount of Zn was also reported. Zn was found in one kohl sample at high concentration (115000 *μ*g g^−1^) corresponding to 11.5% (w/w) of the product [26]. The kohl sample was the same product which contained 950 *μ*g g^−1^ of Cu [26]. Al-Dayel et al. [29] analyzed 9 brands of the most expensive brands names of mascara and eyeshade from the Saudi market. Twenty-eight elements were determined by ICP-MS and flow injection mercury system (FIMS). The highest Zn concentration was 2000 *μ*g g^−1^ in one of the eyeshades. In several facial cosmetics, Zn concentrations of 94.4, 111.9, and 94.9 *μ*g g^−1^ have been reported in eye liners, eye pencils, and lipsticks purchased from open market in Nigeria [30].

Due to the high levels of heavy metals found in kohl products which are widely used as traditional cosmetic in the Tunisian population, strict legislation must be established to ban the use of Pb and Cd as ingredients in the kohl formula. As for consumers, it is recommended not to purchase unlabeled products and to be selective while purchasing cosmetics.

### 3.2. Heavy Metal Contents in Henna Samples

Based on the overall mean concentrations, the mean heavy metal contents in henna products were in the following decreasing order: Zn > Cd > Pb > Cu.

Pb levels in henna ranged from 1.2 to 6.7 *μ*g g^−1^ with an overall mean concentration of 4.3 ± 0.4 *μ*g g^−1^. Pb mean concentrations found in both locally manufactured and imported brands were similar. In comparison to the literature, our results were close to the results found by Bernth et al. [26] about 17 henna products purchased from retail and ethnic shops in Denmark and analyzed by ICP-MS. Pb was found along other heavy metals in 10 out of the 17 analyzed products. The concentrations of lead in 10 of the analyzed products ranged from 0.5 *μ*g g^−1^ to 2.0 *μ*g g^−1^. Paraphenylene diamine (PPD) was found in 3 of the 17 products. The lowest PPD concentration was 0.003% (w/w), while the highest content was 17% (w/w) corresponding to 170 g kg^−1^. Al-Saleh and Coate [31] analyzed Pb in 20 henna products obtained from the local herbalist in the Riadh market in Saudi Arabia by GFAAS. Pb mean concentrations ranged from 1.29 to 16.48 *μ*g g^−1^ with the highest Pb concentration found in a black henna sample imported from Sudan. Pb was also determined by Jallad and Espada-Jallad [32] in 12 commercial and traditional henna samples obtained from local consumer products' superstores in Sharjah and Dubai in the United Arab Emirates. The concentrations found in henna ranged from 2.29 *μ*g g^−1^ to 65.98 *μ*g g^−1^. The highest lead concentration was found in a local black henna paste. Lekouch et al. [23] found that the average concentrations of Pb in 20 henna samples obtained from local herbalists in the Marrakech market ranged from 2.2 to 6.5 *μ*g g^−1^. The two highest Pb levels were recorded in two local black henna samples at concentration of 15.5 and 19.9 *μ*g g^−1^.

As for Cd, the levels ranged from 1.1 to 12.9 *μ*g g^−1^ in the local brands (group 1) and from 0.8 to 18.6 *μ*g g^−1^ in imported products (group 2) with the highest Cd level found in henna products from Yemen and Lybia (H-9 and H-10).

The obtained results were higher than those reported by Bernth et al. [26] where Cd was detected in only four henna samples at concentrations ranging from 0.05 to 0.07 *μ*g g^−1^.

In the present study, Zn levels ranged from 3.7 to 90 *μ*g g^−1^ with the highest Zn concentration found in henna product from Lybia (H-10).

Alwakeel [33] analyzed 5 kinds of henna available in herb markets around the city of Riadh in Saudi Arabia along with 27 samples of well-known herbs. The average mean concentrations ranged from 0.307 to 0.607 *μ*g g^−1^ for Zn and from 0.088 to 0.178 *μ*g g^−1^ for Cu.

The screening for heavy metals in 17 samples of henna conducted by Bernth et al. [26] detected Zn at concentrations ranging from 0.55 to 76 *μ*g g^−1^.

As for Cu levels in henna samples, results ranged between 0.5 and 3.3 *μ*g g^−1^ in local brands and between 0.7 and 1.2 *μ*g g^−1^ in imported products (group 2). These results were close to those found by Bernth et al. [26] where Cu mean concentrations ranged between 0.14 and 12 *μ*g g^−1^.

One possible explanation of the data disparity in literature regarding heavy metals contents between henna brands is the difference in the way of manufacturing the product (different chemical coloring additives) and the product origin. In fact, henna thrives outside arid regions. The countries of origin of this powder are several in number with very different climates. Thereby, the chemical constitution could be variable [34].

In Tunisia, henna plants are cultivated in the region of Gabes located in the southeastern part of Tunisia. This region is well-known for phosphoric acid industries that generate tons of phosphogypsum. This by-product rich with Cd has contaminated the soil and the groundwater of the region for decades. So, Cd among other metals can accumulate in plant tissues at concentrations above the threshold levels believed to threaten the health of human beings.

Henna like kohl is widely used as a traditional cosmetic for hair care and hair dyeing as an alternative to permanent chemical hair dyeing. Henna leaves are picked, dried, grinded to powder, then mixed, and stirred to paste before being massaged into the dry hair and the skull of the head or used as a tattoo on the skin. The red brownish color of henna is derived from a natural substance called lawsone. Other color nuances could be obtained by mixing henna powder with other ingredients such as citrus, pomegranate peel, and gall of* Tamarix orientalis*. Some other chemicals could be added to alter the color of henna including synthetic dye, solvents, and metal salts. Metal salts can interact with the other chemicals, oil, and wax and cause health problems. The most frequently used metal salt in henna was Pb acetate [35]. Some henna pastes have been also noted to include silver nitrate, carmine, pyrogallol, disperse orange dye, and chromium [36]. Black henna has been reported to contain considerable amounts of PPD used to enhance its dark color [36]. In North Africa, especially in the Maghreb region, there are many varieties of henna depending on the traditional way of preparing this cosmetic product. The major difference is in the substances added to henna. Various mineral products rich in trace metals can be mixed with henna such as mercury (zawaq), copper oxide (hadida), zinc oxide (Tûtiya), and litharge PbO (limrataq) [23]. In the present study, all the analyzed henna samples were of green powder except for one local brand sample H-2 where henna color was light red brownish.

Although the quantitative analysis of Pb, Cd, Zn, and Cu in this sample “H2” showed concentrations at the same levels as the other types of henna, the association between its color and its trace metal contents has been further investigated. Since the EDXRF can give a clear indication of the elemental composition, it was used to test the presence of any significant metals that could explain the red brownish color of the henna sample “H-2.” The EDXRF detected Pb, Cd, Zn, and Cu at trace levels (<0.1% weight of the sample) in both green henna sample (H-1) and red-brown henna sample (H-2). Calcium (CaO) and silicon (SiO_2_) were also identified as major components in the EDXRF spectra of both green henna (H1) and red-brown henna (sample H-2). The presence of SiO_2_ in henna has been also reported by Jallad and Espada-Jallad [32] in both red and black paste henna products from the Emirates. SiO_2_ (silica) was reported to be a main constituent of the horsetail extract powder derived from the plant* Equisetum hyemale* and which was mixed with henna powder and used to stimulate, heal, and soften both hair and skin.

**Figure 2:**
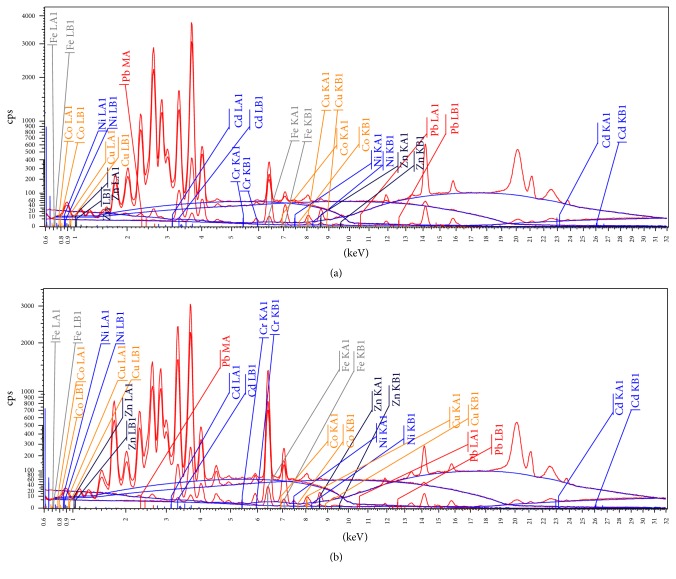
EDXRF spectra of both the (a) green henna sample (H-1) and of the (b) red-brown henna (H-2) sample.

As for the presence of other metal salts in henna, iron, manganese, nickel, and cobalt have been identified at low levels (<0.05%). The comparison between both EDXRF spectra for green (Figure 2(a)) and red-brown henna (Figure 2(b)) showed similar patterns and their elemental composition in terms of heavy metals was similar (Table 4) though Fe levels were higher in red henna (6.66%) than in green one (2.38%). However, the difference between the two products was not significant for any certain attribution of the red-brown colors to the presence of metal salts. Further investigations should be carried out to unveil the origin of this red color.

### 3.3. Toxicity of Heavy Metal

We can note that, in most of the analyzed products, Pb and Cd contents exceed the limits of 20 and 10 *μ*g g^−1^ set by the US Food and Drug Administration (FDA) as maximum amounts of Pb and Cd allowed in color additives used to make cosmetic for external use produced in good manufacturing practices. Taking into consideration its toxicology, kohl is not allowed in USA nor is it allowed to be used in cosmetics [15, 16]. Unfortunately, there are no current international standards for impurities in cosmetics. The Canadian regularity limits for certain metals in cosmetics are 10 *μ*g g^−1^ for Pb, 3 *μ*g g^−1^ for As, Cd, and Hg, and 5 *μ*g g^−1^ for Sb [37]. Other legislations like the Danish Statutory Order on Cosmetics number 489 [38] have banned the marketing of all cosmetic products containing Pb, Cd, Sb, As, Ba, Cd, Cr, Tl, Ni, Co, and potassium bromine. Rather than taking a risk-based approach, the German limits are based on levels that could be technically avoided. Thus, heavy metal impurities are limited to anything above normal background levels. Accordingly, Pb and Cd content in cosmetic products at concentrations above 20 *μ*g g^−1^ and 5 *μ*g g^−1^, respectively, are considered technically avoidable [39].

As for Zn levels in cosmetics, the FDA has classified zinc oxide as category I ingredient for use in cosmetic products as UV filter in concentrations of up to 25% (w/w) [40] while the Scientific Committee on Cosmetics and Non-Food Products (SCCNFP) intended for consumers required more information to enable a proper safety evaluation of micronised zinc oxide for use as UV filter in cosmetic products including possible pathways of cutaneous penetration and systemic exposure [41]. As for the Japanese Ministry of Health and Welfare limits of 1% and 0.5% of the product weight have been adopted as maximum amounts of zinc and copper substituted zeolite in cosmetic not used for mucosa [42].

A daily application of cosmetic products with considerable amounts of Pb, Cd, Zn, and Cu on the skin such as the present products marketed in Tunisia can potentially add up to significant exposure levels. The skin absorption of heavy metals salts varies greatly with different physical parameters. In fact, heavy metals bind with the proteins of the cell by forming complexes with carboxylic acid (–COOH), amine (–NH_2_), and thiol (–SH), hindering their functions and causing death of the cells which lead to multiple diseases [43]. The elevated levels of Pb found in the commercialized kohl samples present a potential risk especially for pregnant women who are more vulnerable consumers as it can easily pass through the placenta. Increased levels of lead in the blood of infants to whom kohl was applied have been well documented [9, 44–47].

In fact, several studies have associated the use of kohl with the elevated blood lead levels in children. A US study showed a significant difference between blood lead levels of children of Pakistani and Indian communities exposed to kohl and those of the same communities not exposed to this product (0.62 *μ*mol L^−1^ versus 0.21 *μ*mol L^−1^) [8]. The poisoning of children by Pb from kohl products could be through oral ingestion as young children tend to rub their eyes when they are irritated by kohl and to put their contaminated fingers in their mouths [12]. Absorption through the nasolacrimal duct or tear duct has also been proposed [8].

The intestinal absorption of Pb is influenced by the type of the ingested derivatives and the gastrointestinal contents and by age. In addition, it is increased in the presence of certain nutritional deficiencies particularly by those of iron, as well as calcium [48].

Also, in adult users of kohl, increased lead blood levels have been observed following its use [27, 49]. Studies of the health effects conducted on kohl showed that adults absorb 5–15% and children can absorb about 41% of the ingested lead [29]. Children under two years of age are particularly vulnerable because the fractions of ingested lead which is absorbed slowly decrease until the age of 2 years and more rapidly, thereafter, approach the adult level absorption around the age of 10 years [48].

Henna has long been used in the countries of the Middle East and North Africa for its cosmetic or therapeutic properties. It has been used for the treatment of certain skin lesions and infected burns. Several of its therapeutic properties have been recently proven. Its anti-inflammatory, antipyretic, analgesic, and even tuberculostatic properties were experimentally demonstrated [50–52]. Despite its low allergic potential, some allergic reactions have been reported. Most of them were delayed-type hypersensitivity reactions and allergic contact dermatitis [23, 53–55].

A toxicological characterization of lawsone, a main bioactive ingredient of henna, was conducted by the European Scientific Committee on Cosmetic Products and Non-Food Products (SCCNFP) intended for consumers in 2004. This latter proposed to consider lawsone as class 2A of dangerous substances due to its potential genotoxicity/mutagenicity [34].

Furthermore, henna is known to be dangerous to people with glucose-6-phosphate dehydrogenase deficiency (G6PD deficiency) which is more common in males than in females [56]. A case of acute poisoning was also reported for a girl aged 15 following a suicidal ingestion of an unknown amount of henna. The autopsy reported laryngeal edema, pulmonary congestion, and pathological disorders related to anaphylaxis [57]. Another case of acute poisoning by henna was also reported for a woman aged 45 following a voluntary ingestion of a liter of henna-based solution prescribed by traditional medicine as a treatment for colitis. Severe bloody diarrhea with abdominal pain and fever was reported [58]. Henna in itself remains a rare and weak skin sensitizer and in most cases allergic reactions were not caused by henna but by the chemical substances added to henna, mainly p-phenylenediamine [54, 55].

In Maghreb countries, this substance is sold by arborist as a rock named “Takouat Roumia.” Being sold over the counter, this highly toxic product has become the preferred substance for suicide attempts. Numerous cases of poisoning and death by this substance have been reported [59].

Metal salts among other ingredients in marketed henna preparations are mixed with different additives to obtain different color shades. The polluted environment in which this plant is cultivated affects also its contents of heavy metals.

The relatively high heavy metal contents found in some henna brands in this present work mainly for Cd, comparing to data from literature, rise serious concerns about possible health problems that daily use of such products can possibly cause, whether through percutaneous absorption or through accidental ingestion. In fact, cadmium is not known to have a physiological function in the human organism, but it is highly toxic even at very low concentrations [60]. An increase level of cadmium has been reported to cause inhibition of DNA mismatches [61] and acute damage to organs such as kidneys, liver, and lungs [60]. Although zinc is not involved in cellular redox cycle and has traditionally been regarded as relatively nontoxic, recent studies increasingly show that free ionic zinc is a potent killer of neurons, glia, and other cells type. Skin contact with zinc powders or concentrated solutions can result in severe corrosive effects including ulceration, blistering, and permanent scarring [62]. Contact dermatitis has been reported in rare instances, following the use of shampoos containing zinc pyrithione but the specific etiological role for zinc was not clear [62]. Zinc has been reported to cause the same signs of illness as does Pb and can be mistakenly diagnosed as Pb poisoning [61]. Copper builds up first in the liver and disrupts the liver's ability to detoxify the blood in general, causing fatal health problems. In addition, Cu is a very stimulating mineral to the nerves and nervous system. Copper toxicity can give rise to many psychological imbalances such as mood swings, depression, anxiety, restlessness, and insomnia [63].

Taking into consideration the relatively high levels of Pb and Cd found in the tested products and their toxicity, an immediate mandatory regular testing program must be conducted to check toxic metals in all the kohl and henna products sold in local markets in order to safeguard the consumer health.

## 4. Conclusions

The data presented in this work provides useful information about Pb, Cd, Cu, and Zn contents in kohl and henna, two most commonly used traditional cosmetic products marketed in Tunisia. Twenty-three items were tested representing twelve henna samples and eleven kohl products.

Some of the products were locally manufactured. The rest were imported mainly from eastern countries. Overall, the study revealed that the heavy metal levels mainly in kohl products were far above the recommended limits. Locally manufactured kohl products contained high amount of Pb and Cd. As for henna samples, considerable amounts of Cd were found in some local brands highlighting the extent of environmental pollution in the southeast of Tunisia. The high contents of heavy metals in some imported brands may evoke the possibility of spuriousness. Despite the sharp drop in lead poisoning among the human population primarily associated with control of exposure sources such as gasoline, paint, and various consumer products, lead exposure remains present through less well identified sources. Thereby, physicians should raise awareness and encourage people to avoid the use of kohl and henna products especially pregnant women unless there is a certainty that they are lead-free products.

Since there are no proper safety regulations in Tunisia, strict legislations must be established to enforce the acceptable limits of potential contaminants in cosmetics and the good manufacturing practice. Regular inspection of marketed products should be also conducted by the concerned authorities to detect any fraudulent or hazardous product. The sale of any unlabeled or noncompliant product should be also prohibited especially for kohl products due to their major risk especially on the health of young children and pregnant women.

## Figures and Tables

**Table 1:** Temperature heating program for graphite furnace atomic absorption spectrophotometer.

Step	Temp. (°C)		Time (s)		Heat mode	Ar flow rate mL·min^−1^
	Pb	Cd	Pb	Cd		
1	170	170	5 (10)	10 (10)	Ramp	250
2	250	250	10 (10)	10 (10)	Ramp	250
3	600 (800)	350 (550)	15 (10)	15 (10)	Ramp	250
4	600 (800)	350 (550)	3 (2)	6 (3)	Step	0
5	1500 (1500)	1700 (1700)	4 (3)	4 (3)	Step	0
6	2400	2400	3	3	Step	250

Tpyr (Pb): 600°C for henna and 800°C for kohl; Tatom (Pb): 1500°C for henna and kohl; Tpyr (Cd): 350°C for henna and 550°C for kohl; Tatom (Cd): 1700°C for henna and kohl.

**Table 2:** Descriptive statistical summary of heavy metal concentration (mean ± SD) in kohl and henna in *µ*g g^−1^.

Product type	Origin	Color	Pb	Cd	Zn	Cu
Kohl (surma)						
	Local					
S1	Tunisia	Black	1954.9 ± 7.62	152.1 ± 4.1	21.0 ± 0.1	82.5 ± 0.1
S2	Tunisia	Black	3759.0 ± 9.83	81.9 ± 5.3	14.5 ± 0.1	52.5 ± 0.2
S3	Tunisia	Black	1504.9 ± 7.62	7.5 ± 0.5	107.5 ± 0.5	162.5 ± 2.1
S4	Tunisia	Black	135.9 ± 1.36	4.4 ± 0.3	102.5 ± 0.2	2.5 ± 0.1
S5	Tunisia	Black	2745.5 ± 19.06	109.7 ± 4.4	137.5 ± 1.0	135 ± 0.1
Mean ± SD (*n* = 3)			2020.0 ± 9.09	71.1 ± 2.9	76.6 ± 0.4	87.0 ± 0.5
	Foreign					
S6	India	Dark black	2483.6 ± 9.85	4.2 ± 0.6	25.0 ± 0.3	3.2 ± 0.1
S7	Pakistan	Dark black	101 ± 1.36	1.0 ± 0.5	112.5 ± 0.5	7.0 ± 0.1
S8	China	Dark Black	240.8 ± 1.48	3.9 ± 0.7	3.5 ± 0.1	6.3 ± 0.1
S9	France	Dark black	51.1 ± 2.1	ND	0.7 ± 0.1	6.7 ± 0.3
S10	India	Dark black	4839.5 ± 4.07	158.6 ± 7.7	185.0 ± 1.5	15.0 ± 0.2
S11	Saudi Arabia	Dark black	3379.9 ± 14.98	71.8 ± 2.4	175.0 ± 1.2	10.0 ± 0.1
Mean ± SD (*n* = 3)			1849.3 ± 5.32	47.9 ± 2.4	83.6 ± 0.6	8.0 ± 0.2
Overall mean ± SD (*n* = 11)			1926.9 ± 7.04	59.5 ± 1.9	80.4 ± 0.5	43.9 ± 0.3
Overall min			**51.1**	**1.0**	**0.7**	**2.5**
Overall max			**4839.5**	**158.6**	**185.0**	**162.5**

Henna						
	Local					
H1	Tunisia	Green	6.7 ± 0.8	5.1 ± 1.1	55.0 ± 0.2	ND
H2	Tunisia	Red/light brown	5.5 ± 0.7	12.9 ± 0.6	47.0 ± 0.2	0.5 ± 0.1
H3	Tunisia	Green	1.2 ± 0.3	5.9 ± 0.2	4.2 ± 0.1	ND
H4	Tunisia	Green	2.7 ± 0.0	1.8 ± 0.1	10.0 ± 0.2	0.5 ± 0.0
H5	Tunisia	Green	3.5 ± 0.6	1.1 ± 0.2	7.5 ± 0.1	3.3 ± 0.1
H6	Tunisia	Green	3.2 ± 0.1	4.8 ± 0.2	12.5 ± 0.1	3.3 ± 0.1
Mean ± SD (*n* = 3)			3.8 ± 0.4	5.3 ± 0.4	22.7 ± 0.2	1.9 ± 0.1
	Foreign					
H7	Sudan	Green	8.9 ± 0.5	4.3 ± 0.9	21.0 ± 0.1	0.7 ± 0.1
H8	India	Green	6.0 ± 0.4	14.9 ± 0.6	15.0 ± 0.1	1.2 ± 0.1
H9	Yemen	Green	5.4 ± 0.1	18.6 ± 0.7	47.5 ± 0.2	ND
H10	Libya	Green	2.6 ± 0.9	18.1 ± 0.8	90.0 ± 0.2	ND
H11	Libya	Green	4.8 ± 0.2	6.9 ± 0.7	3.7 ± 0.1	0.7 ± 0.0
H12	Pakistan	Green	1.8 ± 0.2	0.8 ± 0.2	6.2 ± 0.2	1.0 ± 0.1
Mean ± SD (*n* = 3)			4.9 ± 0.4	10.6 ± 0.6	30.6 ± 0.2	0.9 ± 0.1
Overall mean ± SD (*n* = 12)			4.3 ± 0.4	7.9 ± 0.5	26.6 ± 0.2	1.4 ± 0.1
Overall min			**1.2**	**0.8**	**3.7**	**0.5**
Overall max			**8.9**	**18.6**	**90.0**	**3.3**

**Table 3:** % of weight elemental composition of local kohl sample (S-5) and natural stone “hajar” kohl sample using EDXRF.

Lead-based local sample (S-5)		A natural stone kohl sample	
Formula	Weight %	Formula	Weight %
Pb	94.09	Pb	96.59
Ca	1.03	Ca	1.19
Si	0.98	Cl	0.67
Cu	0.38	P	0.63
Cl	0.29	S	0.18
P	0.27	Cd	0.06
As, Fe	0.26	Fe	0.03
Zn	0.23	Ti	0.20
Al	0.14	Zn	0.02
Cd	0.08	Sn, Sb	0.01
Sb	0.05	Cu, Ni, Co, Cr	<0.01
Ni, Co, Cr	<0.01		

**Table 4:** % of weight composition of local red henna sample (H-2) and green henna sample (H-1) using EDXRF.

Local red Henna sample (H-2)		Local green Henna sample (H-1)	
Formula	Weight %	Formula	Weight %
Na	37.50	Ca	33.19
Ca	17.09	Na	22.30
K	12.59	K	13.77
Si	11.61	Cl	6.47
Fe	6.66	S	6.07
Al	4.54	Mg	5.80
S	3.59	Si	4.26
Cl	2.41	P	2.46
P	1.58	Fe	2.38
Mn	0.44	Al	1.42
Zn	0.08	Mn	0.11
As, Cd	0.04	Zn	0.07
Pb, Cu, Cr, Ni, Co	<0.01	Cu, Sb, Sn	0.04
		Cd	0.03
		Pb	0.02
		As, Ni, Co, Cr	<0.01
